# Ten quick tips for machine learning in computational biology

**DOI:** 10.1186/s13040-017-0155-3

**Published:** 2017-12-08

**Authors:** Davide Chicco

**Affiliations:** Princess Margaret Cancer Centre, PMCR Tower 11-401, 101 College Street, Toronto, Ontario, M5G 1L7 Canada

**Keywords:** Tips, Machine learning, Computational biology, Biomedical informatics, Health informatics, Bioinformatics, Data mining, Computational intelligence

## Abstract

Machine learning has become a pivotal tool for many projects in computational biology, bioinformatics, and health informatics. Nevertheless, beginners and biomedical researchers often do not have enough experience to run a data mining project effectively, and therefore can follow incorrect practices, that may lead to common mistakes or over-optimistic results. With this review, we present ten quick tips to take advantage of machine learning in any computational biology context, by avoiding some common errors that we observed hundreds of times in multiple bioinformatics projects. We believe our ten suggestions can strongly help any machine learning practitioner to carry on a successful project in computational biology and related sciences.

## Introduction

Recent advances in high-throughput sequencing technologies have made large biological datasets available to the scientific community. Together with the growth of these datasets, internet web services expanded, and enabled biologists to put large data online for scientific audiences.

As a result, scientists have begun to search for novel ways to interrogate, analyze, and process data, and therefore infer knowledge about molecular biology, physiology, electronic health records, and biomedicine in general. Because of its particular ability to handle large datasets, and to make predictions on them through accurate statistical models, machine learning was able to spread rapidly and to be used commonly in the computational biology community.

A machine learning algorithm is a computational method based upon statistics, implemented in software, able to discover hidden non-obvious patterns in a dataset, and moreover to make reliable statistical predictions about similar new data. As explained by Kevin Yip and colleagues: “The ability [of machine learning] to automatically identify patterns in data [...] is particularly important when the expert knowledge is incomplete or inaccurate, when the amount of available data is too large to be handled manually, or when there are exceptions to the general cases” [1]. This is clearly the case for computational biology and bioinformatics.

Machine learning (often termed also *data mining*, *computational intelligence*, or *pattern recognition*) has thus been applied to multiple computational biology problems so far [2–5], helping scientific researchers to discover knowledge about many aspects of biology. Despite its importance, often researchers with biology or healthcare backgrounds do not have the specific skills to run a data mining project. This lack of skills often makes biologists delay or decide not to try to include any machine learning analysis in it. In other cases, biological and healthcare researchers who embark on a machine learning venture sometimes follow incorrect practices, which lead to error-prone analyses, or give them the illusion of success.

To avoid those situations, we present here ten quick tips to take advantage of machine learning in any computational biology project. Ten best practices, or ten pieces of advice, that we developed especially for machine learning beginners, and for biologists and healthcare scientists who have limited experience with data mining.

We organize our ten tips as follows. At the beginning, the first five tips regard practices to consider *before* commencing to program a machine learning software (the dataset check and arrangement in Tip 1, the dataset subset split in Tip 2, the problem category framing in Tip 3, the algorithm choice in Tip 4, and the handling of imbalanced dataset problem in Tip 5). After them, the next two tips regard relevant practices to adopt *during* the machine learning program development (the hyper-parameter optimization in Tip 6, and the handling of the overfitting problem in Tip 7). Moreover, the following tip refers to what to do *at the end* of a machine learning algorithm execution (the performance score evaluation in Tip 8). Finally, the last two tips regard broad general best practices on how to arrange a project, and are valid not only in machine learning and computational biology, but in any scientific field (choosing open source programming platforms in Tip 9, and asking feedback and help from experts in Tip 10).

To beginners, the understanding of these ten quick tips should not replace the study of machine learning through a book. On the contrary, we wrote this manuscript to provide a complementary resource to a classical training from a textbook [2], and therefore we suggest all the beginners to start from there.

In this paper, we consider an input dataset for a binary classification task represented as a typical table (or matrix) having *M* data instances as rows, *N* features as columns, and a binary target-label column. Of course, switching the rows with the columns would not change the results of a machine learning algorithm application. We call *negative data instance* a row of the input table with *negative*, *false*, or 0 as target label, and *positive data instance* a row of the input table with *positive*, *true*, or 1 as target label.

Carrying a machine learning project to success might be troublesome, but these ten quick tips can help the readers at least avoid common mistakes, and especially avoid the dangerous illusion of inflated achievement.

## Tip 1: Check and arrange your input dataset properly

Even though it might seem surprising, the most important key point of a machine learning project does not regard machine learning: it regards your dataset properties and arrangement.

First of all, before starting any data mining activity, you have to ask yourself: do I have enough data to solve this computational biology problem with machine learning? Nowadays, in the *Big Data* era, with very large biological datasets publically available online, this question might appear irrelevant, but it really raises an important problem in the statistical learning community and domain. While gathering more data can always be beneficial for your machine learning models [6, 7], deciding what is the minimum dataset size to be able to train properly a machine learning algorithm might be tricky. Even if sometimes this not possible, the ideal situation would be having at least ten times as many data instances as there are data features [8, 9].

After addressing the issue of the dataset size, the most important priority of your project is the dataset arrangement. In fact, the way you engineer your input features, clean and pre-process your input dataset, scale the data features into a normalized range, randomly shuffle the dataset instances, include newly constructed features (if needed) will determine if your machine learning project will succeed or fail in its scientific task. As Pedro Domingos clearly affirmed, in machine learning: “[Dataset] feature engineering is the key” [6].

This advice might seem counter-intuitive for machine learning beginners. In fact, newcomers might ask: how could the success of a data mining project rely primarily on the dataset, and not on the algorithm itself? The explanation is straightforward: popular machine learning algorithms have become widespread, first of all, because they work quite well. Similarly to what Isaac Newton once said, if we can progress further, we do it *by standing on the shoulders of giants*, who developed the data mining methods we are using nowadays. And since these algorithms work so well, and we have plenty of open source software libraries which implement them (Tip 9), we usually do not need to invent new machine learning techniques when starting a new project.

On the contrary, each dataset is unique. Indeed, each dataset has domain-specific features, contains data strictly related to its scientific area, and might contain mistaken values hardly noticeable by inexperienced researchers. The Gene Ontology annotation (GOA) database [10], for example, despite its unquestionable usefulness, has several issues. Since not all the annotations are supervised by human curators, some of them might be erroneous; and since different laboratories and biological research groups might have worked on the same genes, some annotations might contain inconsistent information [11]. Problems like these can strongly influence the performance of a machine learning method application.

Given the importance and the uniqueness of each dataset domain, machine learning projects can succeed only if a researcher clearly understands the dataset details, and he/she is able to arrange it properly before running any data mining algorithm on it. In fact, successful projects happen only when machine learning practitioners work by the side of domain experts [6]. This is particularly true in computational biology.

Arranging a biological dataset properly means multiple facets, often grouped all together into a step called *data pre-processing*.

First, an initial common useful practice is to always randomly shuffle the data instances. This operation removes any possible trend related to the order the data instances were collected, and which might wrongly influence the learning process.

**Fig. 1:**
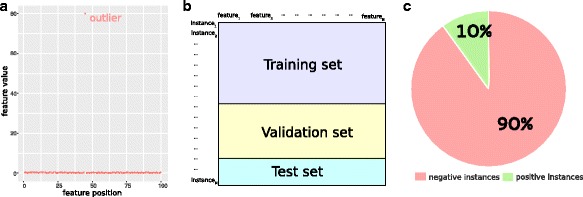
**a** Example of dataset feature which needs data pre-processing and cleaning before being employed in a machine learning program. All the feature data have values in the [0;0.5], except an outlier having value 80 (Tip 1). **b** Representation of a typical dataset table having *N* features as columns and *M* data instances as rows. An effective ratio for the split of an input dataset table: 50% of the data instances for the training set; 30% of the data instances for the validation set; and the last 20% of the data instances for the test set (Tip 2). **c** Example of a typical biological imbalanced dataset, which can contain 90% negative data instances and only 10% positive instances. This aspect can be tackled with *under-sampling* and other techniques (Tip 5)

Moreover, another necessary practice is *data cleaning*, that is discarding all the data which have corrupt, inaccurate, inconsistent, or outlier values [12]. This operation involves expertise and “folk wisdom”, and has to be done carefully. Therefore, we recommend to do it only in the evident cases. Suppose, for example, in a dataset of 100 data instances, you have a particular feature showing values in the [0;0.5] range for 99 instances, and a 80 value for only one single instance (Fig. 1 a). That value is clearly an outlier, and it might be caused by a malfunctioning of the machinery which generated the dataset. Its inclusion in the machine learning phase processing might cause the algorithm to incorrectly classify or to fail to correctly learn from data instances. In this case, you would better remove that particular data element and apply your machine learning only to the remaining dataset, or round that data value to the upper limit value among the other data (0.5 in this case). When handling a large dataset, removing the outliers is the best plan, because you still have enough data to train your model properly. When the dataset size is small-scale and each data instance is precious, instead, it is better to round the outliers to the maximum (or minimum) limit.

For numerical datasets, in addition, the *normalization* (or *scaling*) by feature (by column) into the [0;1] interval is often necessary to put the whole dataset into a common frame, before the machine learning algorithm process it. Latent semantic indexing (LSI), for example, is an information retrieval method which necessitates this pre-processing to be employed for prediction of gene functional annotations [13]. Data normalization into the [*min*;*max*] interval, or into an interval having a particular mean (for example, 0.0) and a particular standard deviation (for example, 1.0) are also popular strategies [14].

An effective advice related to *data pre-processing*, finally, is always to start with a small-scale dataset. In biology, it is common to have large datasets made of millions or billions of instances. So, if you have a large dataset, and your machine learning algorithm training lasts days, create a small-scale miniature dataset with same positive/negative ratio of the original, in order to reduce the processing time to few minutes. Then use that synthesized limited dataset to test and adjust your algorithm, and keep it separated from the original large dataset. Once the algorithm is generating satisfying results on the synthesized toy dataset, apply it to the original large dataset, and proceed.

## Tip 2: Split your input dataset into *three* independent subsets (*training set*, *validation set*, *test set*), and use the *test set* only once you complete training and optimization phases

Many textbooks and online guides say machine learning is about splitting the dataset in two: training set and test set. This approach is incomplete, since it does not take into account that almost always your algorithm has a few key hyper-parameters to be selected before applying the model (Tip 6).

In fact, a common mistake in machine learning is using, in the test set, data instances already used during the training phase or the hyper-parameter optimization phase, and then obtaining inflated performance scores [15]. But as Richard Feynman used to say, in science and in life: “The first principle is that you must not fool yourself, and you are the easiest person to fool”.

Therefore, to avoid *hallucinating* yourself this way, you should always split your input dataset into three independent subsets: *training set*, *validation set*, and *test set*. A common suggested ratio would be 50% for the *training set*, 30% for the *validation set*, and 20% for the *test set* (Fig. 1 b). When dataset is too small and this split ratio is not possible, machine learning practitioners should consider alternative techniques such as *cross-validation* [16] (Tip 7).

After the subset split, use the training set and the validation set to train your model and to optimize the hyper-parameter values, and withhold the test set. Do not touch it. Finally, at the very end, once you have found the best hyper-parameters and trained your algorithm, apply the trained model to the test set, and check the performance results.

This approach (also termed the “lock box approach” [17]) is pivotal in every machine learning project, and often means the real difference between success and failure.

In fact, as Michael Skocik and colleagues [17] noticed, setting aside a subset and using it only when the models are ready is an effective common practice in machine learning competitions. The authors of that paper, moreover, suggest that all the machine learning projects in neuroscience routinely incorporate a lock box approach. We agree and revamp this statement: the lock box approach should be employed by every machine learning project in every field.

## Tip 3: Frame your biological problem into the right algorithm category

You have your biological dataset, your scientific question, and a scientific goal for your project. You have arranged and engineered your dataset, as explained in Tip 1. You decide you want to solve your scientific project with machine learning, but you are undecided about what algorithm to start with.

Before choosing the data mining method, you have to frame your biological problem into the right algorithm category, which will then help you find the right tool to answer your scientific question.

Some key questions can help you understand your scientific problem. Do you have labeled targets for your dataset? That is, for each data instance, do you have a ground truth label which can tell you if the information you are trying to identify is associated to that data instance or not? If yes, your problem can be attributed to the *supervised learning* category of tasks, and, if not, to the the *unsupervised learning* category [4].

For example, suppose you have a dataset where the rows contain the profiles of patients, and the columns contain biological features related to them [18]. One of the features states the diagnosis of the patient, that is if he/she is *healthy* or *unhealthy*, which can be termed as *target* (or *output variable*) for this dataset. Since, in this case, the dataset contains a target label for each data instance, the problem of predicting these targets can be named *supervised learning*. Popular supervised learning algorithms in computational biology are support vector machines (SVMs) [19], *k*-nearest neighbors (*k*-NN) [20], and random forests [21].

If the target can have a finite number of possible values (for example, *extracellular*, or *cytoplasm*, or *nucleus* for a specific cell location), we call the problem *classification* task. And if the possible target values are only two (like *true* or *false*, 0 or 1, *healthy patient* or *unhealthy patient*), we name it *binary classification*.

If the targets are real values, instead, the problem would be named *regression* task.

Target labels are not always present in biological datasets. When data are unlabeled, machine learning can still be employed to infer hidden associations between data instances, or to discover the hidden structure of a dataset. These cases are called *unsupervised learning*, or *cluster analysis* tasks. Common unsupervised learning methods in computational biology include *k*-means clustering [22], truncated singular value decomposition (SVD) [23], and probabilistic latent semantic analysis (pLSA) [24].

Once you studied and understood your dataset, you have to decide to which of these categories of problems you should address your project, and then you are ready to choose the proper machine learning algorithm to start your predictions.

## Tip 4: Which algorithm should you choose to start? The simplest one!

Once you understand what kind of biological problem you are trying to solve, and which method category can fit your situation, you then have to choose the machine learning algorithm with which to start your project. Even if it always advisable to use multiple techniques and compare their results, the decision on which one to start can be tricky.

Many textbooks suggest to select a machine learning method by just taking into account the problem representation, while Pedro Domingos [6] suggests to take into account also the cost evaluation, and the performance optimization.

This algorithm-selection step, which usually occurs at the beginning of a machine learning journey, can be dangerous for beginners. In fact, an inexperienced practitioner might end up choosing a complicated, inappropriate data mining method which might lead him/her to bad results, as well as to lose precious time and energy. Therefore, this is our tip for the algorithm selection: if undecided, start with the simplest algorithm [25].

By employing a simple algorithm, you will be able to keep everything under control, and better understand what is happening during the application of the method. In addition, a simple algorithm will provide better generalization skills, less chance of overfitting, easier training and faster learning properties than complex methods.

Examples of *simple* algorithms are *k*-means clustering for unsupervised learning [22] and *k*-nearest neighbors (*k*-NN) for supervised learning [26]. Even though stating the level of *simplicity* of a machine learning method is not an easy task, we consider *k*-means and *k*-NN *simple* algorithms because they are easier to understand and to interpret than other models, such as artificial neural networks [27] or support vector machines [19].

Regarding *k*-NN, suppose for example you have a complementary DNA (cDNA) microarray input dataset made of 1,000 real data instances, each having 80 features and 1 binary target label. This dataset can be represented with a table made of 1000 rows and 81 columns. Following our suggestion, if you think that your biological dataset can be *learnt* with a *supervised learning* method (Tip 3), you might consider to begin to classify instances with simple algorithm such as *k*-nearest neighbors (*k*-NN) [26]. This method assigns each new observation (an 80-dimension point, in our case) to the class of the majority of *k*-nearest neighbors (the *k* nearest points, measured with Euclidean distance) [28].

Consequently, given the simplicity of the algorithm, you will be able to oversee (and to possibly debug) each step of it, especially if problems arise. In case you reach a satisfying performance with *k*-nearest neighbors, you will be able to stick with it, and proceed in your project. Otherwise, you will always be able to switch to another algorithm, and employ the *k*-nearest neighbors results for a baseline comparison.

As David J. Hand explained, complex models should be employed only if the dataset features provide some reasonable justification for their usage [25].

## Tip 5: Take care of the imbalanced data problem

In computational biology and in bioinformatics, it is often common to have *imbalanced* datasets. An imbalanced (or unbalanced) dataset is a dataset in which one class is over-represented respect to the other(s) (Fig. 1 c). For example, a typical dataset of Gene Ontology annotations, that can be analyzed with a non-negative matrix factorization, usually has only around 0.1% of positive data instances, and 99.9% of negative data instances [11, 23].

In these common situations, the dataset ratio can be a problem: how can you train a classifier to be able to correctly predict both *positive* data instances, and *negative* data instances, if you have such a huge difference in the proportions? Probably, your learning model is going to learn fast how to recognize the over-represented *negative* data instances, but it is going to have difficulties recognizing the scarce subset instances, that are the *positive* items in this case.

Our heuristic suggestion on what ratio of elements to use in the training set is to pick up the average value between 50% and the real proportion percentage. Therefore, in the 90%:10% example, insert in your training set (90*%*+50*%*)/2=70*%* negative data instances, and (10*%*+50*%*)/2=30*%* positive data instances. Obviously, this procedure is possible if there are enough data for each class to create a 70%:30% training set. Alternatively, you can balance the dataset by incorporating the empirical label distribution of the data instances, following Bayes’ rule [29]. Even if more precise, this strategy might be too complicated for beginners; this is why we suggest to use the afore-mentioned heuristic ratio to start.

In addition, there are multiple effective techniques to handle the imbalanced data problem [30]. The best way to tackle this problem is always to collect more data.

If this is not possible, a common and effective strategy to handle imbalanced datasets is the *data class weighting*, in which different weights are assigned to data instances depending if they belong to the majority class or the minority class [31]. Data class weighting is a standard technique to fight the imbalanced data problem in machine learning.

An alternative method to deal with this issue is *under-sampling* [32], where you just remove data elements from the over-represented class. The disadvantage here is that you do not let the classifier learn the excluded data instances.

In addition, other techniques exist, even if trying the aforementioned ones first might be already enough for your machine learning project [30]. Moreover, to properly take care of the imbalanced dataset problem, when measuring your prediction performances, you need to rely not on *accuracy* (Eq. 1), *balanced accuracy* [33], or *F1 score* (Eq. 2), but rather on the *Matthews correlation coefficient* (MCC, Eq. 3). As we will better explain later (Tip 8), among the common performance evaluation scores, MCC is the only one which correctly takes into account the ratio of the confusion matrix size. Especially on imbalanced datasets, MCC is correctly able to inform you if your prediction evaluation is going well or not, while *accuracy* or *F1 score* would not.

## Tip 6: Optimize each hyper-parameter

**Fig. 2:**
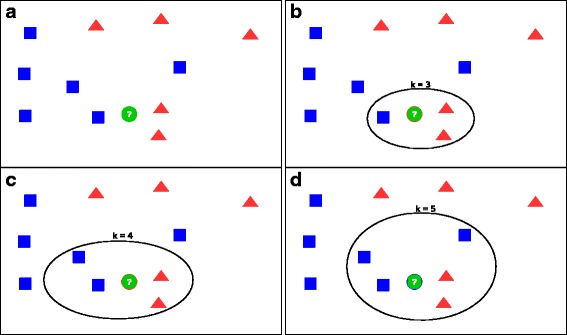
Example of how an algorithm’s behavior and results change when the hyper-parameter changes, for the the *k*-nearest neighbors method [20] (image adapted from [72]). **a** In this example, there are six blue square points and five red triangle points in the Euclidean space. A new point (the green circle) enters the space, and *k*-NN has to decide to which category to assign it (red triangle or blue square). **b** If we set the hyper-parameter *k*=3, the algorithm considers only the three points nearest to the new green circle, and assigns the green circle to the red triangle category (two red triangles versus one blue square). **c** Likewise, if we set the hyper-parameter *k*=4, the algorithm considers only the four points nearest to the new green circle, and assigns the green circle again to the red triangle category (the two red triangles are nearer to the green circle than the two blue squares). **d** However, if we set the hyper-parameter *k*=5, the algorithm considers only the five points nearest to the new green circle, and assigns the green circle to the blue square category (three blue squares versus two red triangles)

The hyper-parameters of a machine learning algorithm are higher-level properties of the algorithm statistical model, which can strongly influence its complexity, its speed in learning, and its application results. Indeed, examples of hyper-parameters are the number *k* of neighbors in *k*-nearest neighbors (Fig. 2) [26], the number *k* of clusters in *k*-means clustering [22], the number of topics (classes) in topic modeling [24], and the dimensions of an artificial neural network (number of hidden layers and number of hidden units) [34]. The hyper-parameters cannot be learned by the algorithm directly from the training phase, and rather they must be set before the training step starts.

A useful practice to select the best suitable value for each hyper-parameter is a grid search. After having divided the input dataset into *training set*, *validation set*, and *test set*, withhold the test set (as explained in Tip 2), and employ the validation set to evaluate the algorithm when using a specific hyper-parameter value. For each possible value of the hyper-parameters, then, train your model on the training set and evaluate it on the validation set, through the *Matthews correlation coefficient* (MCC) or the Precision-Recall area under the curve (Tip 8), and record the score into an array of real values. Once you have tried all the possible values of hyper-parameters, choose the one which led to the highest performance score (*best*_*h*_ in Algorithm 1). Finally, train the model having *best*_*h*_ as hyper-parameter on the training set, and apply it to the test set (Algorithm 1).



Alternatively, you can consider taking advantage of some *automatic machine learning* software methods, which automatically optimize the hyper-parameters of the algorithm you selected. These packages include Auto-Sklearn [35], Auto-Weka [36], TPOT [37], and PennAI [38].

Once again, we want to highlight the importance of the splitting the dataset into three different independent subsets: training set, validation set, and test set. These three subsets must contain no common data instances, and the data instances must be selected randomly, not to make the data collection order influence the algorithm. For these reasons, we strongly suggest to apply a randomly shuffle to the whole input dataset, just after the dataset reading (first line of Algorithm 1).

## Tip 7: Minimize overfitting

As Pedro Domingos correctly stated: “Overfitting is the bugbear of machine learning” [6]. In data mining, overfitting happens every time an algorithm excessively adapts to the training set, and therefore performs badly in the validation set (and test set).

Overfitting happens as a result of the statistical model having to solve two problems. During training, it has to minimize its performance error (often measured through mean square error for regression, or cross-entropy for classification). But during testing, it has to maximize its skills to make correct predictions on unseen data. This “double goal” might lead the model to *memorize* the training dataset, instead of *learning* its data trend, which should be its main task.

Fortunately, there are a few powerful tools to battle overfitting: *cross-validation*, and *regularization*. In 10-fold cross-validation, the statistical model considers 10 different portions of the input dataset as training set and validation set, in a series. After shuffling the input dataset instances and setting apart the test set, the algorithm takes the remaining dataset and divides it into ten folds. It then creates a loop for *i* going from 1 to 10. For each iteration, the cross validation sets the data of the *i*_*th*_ fold as validation set, then trains the algorithm on the remaining dataset folds, and finally applies the algorithm to the validation set. To measure the performance of the classifier in this phase, the user can estimate the median variance of the predictions made in the 10-folds. The algorithm designer can choose a number of *k* folds different from 10, even if 10 is a heuristic common choice that allows the training set to contain the 90% of the data instances and the validation set to contain the 10%.

With *cross-validation*, the trained model does not overfit to a specific training subset, but rather is able to learn from each data fold, in turn.

In addition, *regularization* is a mathematical technique which consists of penalizing the evaluation function during training, often by adding penalization values that increase with the weights of the learned parameters [39].

In conclusion, as any machine learning expert will tell you, overfitting will always be a problem for machine learning. But the awareness of this problem, together with the aforementioned techniques, can effectively help you to reduce it.

## Tip 8: Evaluate your algorithm performance with the Matthews correlation coefficient (MCC) or the Precision-Recall curve

When you apply your trained model to the validation set or to the test set, you need statistical scores to measure your performance.

In fact, in a typical supervised binary classification problem, for each element of the validation set (or test set) you have a label stating if the element is *positive* or *negative* (1 or 0, usually). Your machine learning algorithm makes a prediction for each element of the validation set, expressing if it is *positive* or *negative*, and, based upon these prediction and the gold-standard labels, it will assign each element to one of the following categories: true negatives (TN), true positives (TP), false positives (FP), false negatives (FN) (Table 1).

**Table 1:** The confusion matrix: each pair (actual value; predicted value) falls into one of the four listed categories

	predicted positive	predicted negative
actual positive	TP (true positives)	FN (false negatives)
actual negative	FP (false positives)	TN (true negatives)

If many elements of the set then fall into the first two classes (TP or TN), this means that your algorithm was able to correctly predict as *positive* the elements that were *positive* in the validation set (TP), or to correctly classify as *negative* the instances that were *negative* in the validation set (TN). On the contrary, if you have many FP instances, this means that your method wrongly classified as *positive* many elements which are *negative* in the validation set. And, as well, many FN elements mean that the classifier wrongly predicted as *negative* a lot of elements which are *positive* in the validation set.

In order to have an overall understanding of your prediction, you decide to take advantage of common statistical scores, such as *accuracy* (Eq. 1), and *F1 score* (Eq. 2).

1 $$ accuracy = \frac{TP+TN}{TP+TN+FP+FN}   $$

(*accuracy*: worst value =0; best value =1)

2 $$ F1 \; score = \frac{2 \cdot TP}{2 \cdot TP+FP+FN}   $$

(*F1 score*: worst value =0; best value =1)

However, even if *accuracy* and *F1 score* are widely employed in statistics, both can be misleading, since they do not fully consider the size of the four classes of the confusion matrix in their final score computation.

Suppose, for example, you have a very imbalanced validation set made of 100 elements, 95 of which are *positive* elements, and only 5 are *negative* elements (as explained in Tip 5). And suppose also you made some mistakes in designing and training your machine learning classifier, and now you have an algorithm which always predicts *positive*. Imagine that you are not aware of this issue.

By applying your only-*positive* predictor to your imbalanced validation set, therefore, you obtain values for the confusion matrix categories:

TP = 95, FP = 5; TN = 0, FN = 0.

These values lead to the following performance scores: *accuracy* = 95%, and *F1 score* = 97.44%. By reading these over-optimistic scores, then you will be very happy and will think that your machine learning algorithm is doing an excellent job. Obviously, you would be on the wrong track.

On the contrary, to avoid these dangerous misleading illusions, there is another performance score that you can exploit: the *Matthews correlation coefficient* [40] (MCC, Eq. 3).

3 $$ MCC = \frac{TP \cdot TN - FP \cdot FN}{\sqrt{(TP+FP)\cdot(TP+FN)\cdot(TN+FP)\cdot(TN+FN)}}   $$

(MCC: worst value =−1; best value =+1).

By considering the proportion of each class of the confusion matrix in its formula, its score is high only if your classifier is doing well on both the *negative* and the *positive* elements.

In the example above, the MCC score would be *undefined* (since TN and FN would be 0, therefore the denominator of Eq. 3 would be 0). By checking this value, instead of *accuracy* and *F1 score*, you would then be able to notice that your classifier is going in the wrong direction, and you would become aware that there are issues you ought to solve before proceeding.

Let us consider this other example. You ran a classification on the same dataset which led to the following values for the confusion matrix categories:

TP = 90, FP = 5; TN = 1, FN = 4.

In this example, the classifier has performed well in classifying *positive* instances, but was not able to correctly recognize *negative* data elements. Again, the resulting *F1 score* and *accuracy* scores would be extremely high: *accuracy* = 91%, and *F1 score* = 95.24%. Similarly to the previous case, if a researcher analyzed only these two score indicators, without considering the MCC, he/she would wrongly think the algorithm is performing quite well in its task, and would have the illusion of being successful.

On the other hand, checking the *Matthews correlation coefficient* would be pivotal once again. In this example, the value of the MCC would be 0.14 (Eq. 3), indicating that the algorithm is performing similarly to random guessing. Acting as an *alarm*, the MCC would be able to inform the data mining practitioner that the statistical model is performing poorly.

For these reasons, we strongly encourage to evaluate each test performance through the *Matthews correlation coefficient* (MCC), instead of the *accuracy* and the *F1 score*, for any binary classification problem.

In addition to the *Matthews correlation coefficient*, another performance score that you will find helpful is the Precision-Recall curve. Often you will not have binary labels (for example, true and false) for *negative* and the *positive* elements in your predictions, but rather a real value of each prediction made, in the [0,1] interval. In this common case, you can decide to utilize each possible value of your prediction as threshold for the confusion matrix.

**Fig. 3:**
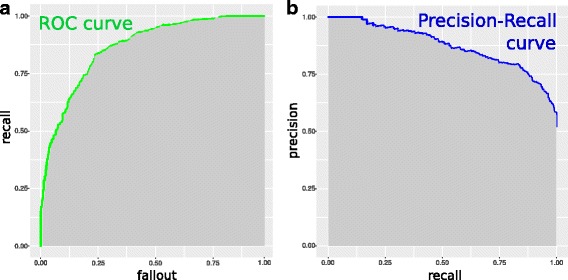
**a** Example of Precision-Recall curve, with the precision score on the y axis and the recall score on the x axis (Tip 8). The grey area is the PR cuve area under the curve (AUPRC). **b** Example of receiver operating characteristic (ROC) curve, with the recall (true positive rate) score on the y axis and the fallout (false positive rate) score on the x axis (Tip 8). The grey area is the ROC area under the curve (AUROC)

Therefore, you will end up having a real valued array for each FN, TN, FP, TP classes. To measure the quality of your performance, you will be able to choose between two common curves, of which you will be able to compute the area under the curve (AUC): *receiver operating characteristic* (ROC) curve (Fig. 3 a), and *Precision-Recall* (PR) curve (Fig. 3 b) [41].

The ROC curve is computed through *recall* (*true positive rate, sensitivity*) on the *y* axis and *fallout* (*false positive rate*, or 1 − *specificity*) on the *x* axis:

*ROC* curve axes:

4 $$ recall = \frac{TP}{TP+FN} \qquad \qquad \qquad fallout = \frac{FP}{FP+TN}  $$

In contrast, the *Precision-Recall* curve has *precision* (*positive predictive value*) on the *y* axis and *recall* (*true positive rate, sensitivity*) on the *x* axis:

*Precision-Recall* curve axes:

5 $$ precision = \frac{TP}{TP+FP} \qquad \qquad \qquad recall = \frac{TP}{TP+FN}   $$

Usually, the evaluation of the performance is made by computing the area under the curve (AUC) of these two curve models: the greater the AUC is, the better the model is performing.

As one can notice, the optimization of the ROC curve tends to maximize the correctly classified *positive* values (TP, which are present in the numerator of the *recall* formula), and the correctly classified *negative* values (TN, which are present in the denominator of the fallout formula).

Differently, the optimization of the PR curve tends to maximize to the correctly classified *positive* values (TP, which are present both in the *precision* and in the *recall* formula), and does not consider directly the correctly classified *negative* values (TN, which are absent both from the *precision* and in the *recall* formula).

In computational biology, we often have very sparse dataset with many *negative* instances and few *positive* instances. Therefore, we prefer to avoid the involvement of *true negatives* in our prediction score. In addition, ROC and AUROC present additional disadvantages related to their interpretation in specific clinical domains [42].

For these reasons, the *Precision-Recall curve* is a more reliable and informative indicator for your statistical performance than the *receiver operating characteristic* curve, especially for imbalanced datasets [43].

Other useful techniques to assess the statistical significance of a machine learning predictions are *permutation testing* [44] and *bootstrapping* [45].

## Tip 9: Program your software with open source code and platforms

When starting a machine learning project, one of the first decisions to take is which programming language or platform you should use. While different packages provide different methods, different execution speed, and different features, we strongly suggest you to avoid proprietary software, and instead to work only with free open source machine learning software packages.

Using proprietary software, in fact, can cause you several troubles. First of all, it limits your collaboration possibilities only to people who have a license to use that specific software. For example, suppose you are working in a hospital, and would like a collaborator from a university to work on your software code. If you are working with a proprietary software, and his/her university does not have the same software license, the collaboration cannot happen. On the contrary, if you use an open source platform, you will not face these problem and will be able to start a partnership with anyone willing to work with you.

Another big problem with proprietary software is that you will not be able to re-use your own software, in case you switch job, and/or in case your company or institute decides not to pay the software license anymore. On the contrary, if you work with open source programs, you will always be able to re-use your own software in the future, even if switching jobs or work places.

For these and other reasons, we advice you to work only with free open source machine learning software packages and platforms, such as R [46], Python [47], Torch [48], and Weka [49].

R is an interpreted programming language for statistical computing and graphics, extremely popular among the statisticians’ community. It provides several libraries for machine learning algorithms (including, for example, *k*-nearest neighbors and *k*-means), effective libraries for statistical visualization (such as ggplot2 [50]), and statistical analysis packages (such as the extremely popular Bioconductor package [51]). On the other hand, Python is a high-level interpreted programming language, which provides multiple fast machine learning libraries (for example, Pylearn2 [52], Scikit-learn [53]), mathematical libraries (such as Theano [54]), and data mining toolboxes (such as Orange [55]). Torch, instead, is a programming language based upon lua [56], a platform, and a set of very fast libraries for deep artificial neural networks. On the other hand, Waikato Environment for Knowledge Analysis (Weka) is a platform for machine learning libraries [49]. Its software is written in Java, and it was developed at the University of Waikato (New Zealand).

For beginners, we strongly suggest starting with R, possibly on an open source operating system (such as Linux Ubuntu). In fact, using open source programming languages and platforms will also facilitate scientific collaborations with researchers in other laboratories or institutions [57].

In addition, we also advise you share your software code publically on the internet, among the publication of your project paper and datasets [58, 59]. In fact, as Nick Barnes explained: “Freely provided working code, whatever its quality, [...] enables others to engage with your research” [60]. Even more, releasing your code openly in the internet also allows the computational reproducibility of your paper results [61].

Together with the usage of open source software, we recommend two other optimal practices for computational biology and science in general: write in-depth documentation about your code [62, 63], and keep a lab notebook about your project [64].

Writing complete documentation for your software and keeping a scientific diary updated about your progress will save a lot of time for your future self, and will be a priceless resource for the success of your project.

## Tip 10: Ask for feedback and help to computer science experts, or to collaborative Q&A online communities

During the progress of a scientific project, asking for a review by experts in the field is always a useful idea. Therefore, if you are a biologist or a healthcare researcher working near a university, surely you should consider contacting a machine learning professional in the computer science department, and ask him/her to meet to gain useful feedback about your project.

Sometimes when meeting a data mining expert in person is not possible, you should then consider to get feedback about your project from data mining professionals through collaborative question-and-answer (Q&A) websites such as Cross Validated, Stack Overflow, Quora, BioStars, and Bioinformatics beta [65].

Cross Validated is a Q&A website of the Stack Exchange platform, mainly for questions related to statistics [66]. Similarly, Stack Overflow is part of the same platform, and it is probably the most-known Q&A website among programmers and software developers [67]. It often includes questions and answers about machine learning software. On the other hand, if Cross Validated and Stack Overflow are more about using users’ interactions and expertise to solve specific issues, you can post broader and more general questions on Quora, whose answers can probably help you better if you are a beginner [68].

Regarding bioinformatics and computational biology, two useful Q&A platforms are BioStars [69, 70] and the recently released Bioinformatics beta [71].

Indeed, the feedback you receive will be priceless: the community users will be able to notice aspects that you did not consider, and will provide you suggestions and help which will make your approach unshakeable.

In addition, many questions and clarifications that the community users ask you will anticipate the possible questions of reviewers of a journal after the submission of your manuscript describing your machine learning algorithm. Finally, your question and its community answers will be able to help other users having the same issues in the future, too.

## Conclusion

Running a machine learning project in computational biology, without making common mistakes and without fooling yourself, can be a hard task, especially if you are a beginner. We believe these ten tips can be an useful checklist of best practices, lessons learned, ways to avoid common mistakes and over-optimistic inflated results, and general pieces of advice for any data mining practitioner in computational biology: following them from the moment you start your project can significantly pave your way to success.

Even though we originally developed these tips for apprentices, we strongly believe they should be kept in mind by experts, too. Nowadays, multiple topics covered by our tips are broadly discussed and analyzed in the machine learning community (for example, overfitting, hyper-parameter optimization, imbalanced dataset), while unfortunately other tip topics are still inadequately uncommon (for example, the usage of *Matthews correlation coefficient*, and open source platforms). With this manuscript, we hope these concepts can spread and become common practices in every data mining project.

## Abbreviations

AUC: Area under the curve
cDNA: Complementary DNA
FN: False negatives
FP: False positives
GO: Gene Ontology
GOA: Gene Ontology annotations
*k*-NN: *k*-nearest neighbors
LSI: Latent semantic indexing
MCC: Matthews correlation coefficient
MSE: Mean square error
PR: Precision-recall
pLSA: Probabilistic latent semantic analysis
Q&A: Questions and answers
ROC: Receiver operating characteristic
SVD: Singular value decomposition
SVM: Support vector machine
TN: True negatives
TP: True positives
